# Assessment of the corpus callosum size in male individuals with high intelligence quotient (members of Mensa International)

**DOI:** 10.1007/s00117-023-01146-3

**Published:** 2023-05-09

**Authors:** Andrzej Urbanik, Wiesław Guz, Marek Gołębiowski, Edyta Szurowska, Agata Majos, Marek Sąsiadek, Marek Stajgis, Monika Ostrogórska

**Affiliations:** 1 Department of Radiology, Collegium Medicum, Jagiellonian University, Kopernika 19, 31-501 Krakow, Poland; 2 Department of Electroradiology, University of Rzeszów, Rzeszów, Poland; 3 I-st Department of Clinical Radiology, Medical University of Warsaw, Warszawa, Poland; 4 2nd Department of Radiology, Medical University of Gdańsk, Gdańsk, Poland; 5 Chair of Radiology and Imaging Diagnostics, Medical University of Łódź, Łódź, Poland; 6 Department of Radiology, Wroclaw Medical University, Wrocław, Poland; 7 Department of General Radiology and Neuroradiology, Poznan University of Medical Sciences, Poznań, Poland

**Keywords:** Brain, Isthmus thickness, Intelligence factors, Magnetic resonance imaging (MRI), Morphological measurements, Gehirn, Dicke des Isthmus, Intelligenzfaktoren, Magnetresonanzbildgebung, Morphologische Messungen

## Abstract

**Objectives:**

The aim of this study was to assess the size of the corpus callosum in members of Mensa International, which is the world’s largest and oldest high-intelligence quotient (IQ) society.

**Methods:**

We performed T2-weighted magnetic resonance imaging (Repetition Time, TR = 3200 ms, Time of Echo, TE = 409 ms) to examine the brain of members of Mensa International (Polish national group) in order to assess the size of the corpus callosum. Results from 113 male MENSA members and 96 controls in the age range of 21–40 years were analyzed.

**Results:**

The comparative analysis showed that the mean length of the corpus callosum and the thickness of the isthmus were significantly greater in the Mensa members compared to the control groups. A statistically significant difference was also identified in the largest linear dimension of the brain from the frontal lobe to the occipital lobe. The mean corpus callosum cross-sectional area and its ratio to the brain area were significantly greater in the Mensa members.

**Conclusions:**

The results show that the dimensions (linear measures and midsagittal cross-sectional surface area) of the corpus callosum were significantly greater in the group of Mensa members than in the controls.

## Abstract (de)

**Ziel:**

Das Ziel der vorliegenden Studie bestand darin, die Größe des Corpus callosum bei Mitgliedern von Mensa International zu bestimmen, der weltgrößten und ältesten Gesellschaft für hochbegabte Menschen.

**Methoden:**

Es wurde eine T2-gewichtete Magnetresonanztomographie (Repetitionszeit, TR: 3200 ms, Echozeit, TE: 409 ms) durchgeführt, um das Gehirn von Mitgliedern von Mensa International (polnische Landesgruppe) zu untersuchen mit dem Ziel, die Größe des Corpus callosum zu ermitteln. Dazu wurden die Ergebnisse von 113 männlichen Mensa-Mitgliedern und 96 Kontrollen mit einer Altersspanne von 21–40 Jahren ausgewertet.

**Ergebnisse:**

In der vergleichenden Auswertung zeigte sich, dass bei den Mensa-Mitgliedern die mittlere Länge des Corpus callosum und die Dicke des Isthmus signifikant größer waren als in der Kontrollgruppe. Eine statistisch signifikante Differenz wurde auch in der größten linearen Ausdehnung des Gehirns vom Frontallappen bis zum Okzipitallappen ermittelt. Die mittlere Querschnittsfläche des Corpus callosum und ihr Verhältnis zu Fläche des Gehirns waren bei Mensa-Mitgliedern ebenfalls signifikant größer.

**Schlussfolgerung:**

Die Ergebnisse zeigen, dass die Abmessungen des Corpus callosum (lineare Maße und mittsagittale Oberfläche im Querschnitt) bei Mensa-Mitgliedern signifikant größer waren als bei den Kontrollen.

## Introduction

Intelligence is a broad and widely disputed concept in psychology used with reference to an individual’s capacities for cognitive performance. According to a definition generally accepted by researchers, “intelligence is a very general mental capability that, among other things, involves the ability to reason, plan, solve problems, think abstractly, comprehend complex ideas, learn quickly and learn from experience. It is not merely book learning, a narrow academic skill, or test-taking smarts. Rather it reflects a broader and deeper capability for comprehending our surroundings—‘catching on,’ ‘making sense’ of things, or ‘figuring out’ what to do” [1]. Individuals vary in terms of the capacities referred to as intelligence, which is why tests have been devised to assess these capabilities [2]. A precursor to the currently used tools, a test designed to assess general intellectual capacities of children was published in 1905 by Alfred Binet and Théodore Simon. In 1912 William Stern proposed a measurable index (intelligence quotient [IQ]) to describe the overall result achieved in intelligence tests [3]. Hence, it became natural to look for correlations between human intelligence and the morphology or physiology of the brain [4].

The earliest attempts of this type focused on the brains of outstanding individuals [5, 6]. Many research centers collected preserved brains of such individuals. At present the largest collection of “cognitively normal” brains is owned by McMaster University in Hamilton (Ontario, Canada). One of the most comprehensively investigated was Albert Einstein’s brain [7]. Particularly notable is the study carried out in Shanghai-based East China Normal University’s Department of Physics [8]. The authors hypothesized that Einstein’s remarkable intelligence could be linked to his corpus callosum (CC), which was thicker (than in the control groups) enabling more efficient communication between the cerebral hemispheres. There are some studies in which associations between cognitive abilities and CC characteristics were examined in samples with neurological conditions, developmental disabilities and disorders, or diseases [9, 10] but only few studies regarding the correlation of IQ and brain structures in healthy individuals [11–16]. However, there is a lack of sufficient research conducted with people who are above average intelligence. In view of this, the present study aimed to assess the size of the CC in members of Mensa, which is the world’s largest and oldest high-IQ society [17, 18]. To the best of the authors’ knowledge, this is the first such study on Mensa members.

## Material and method

The project was approved by the local bioethics commission (approval no. 122.6120.28.2017). Magnetic resonance imaging (MRI) was applied to examine the brain of 113 male right-handed members of Mensa International (Polish national group) in order to assess the size of the CC. As defined by Mensa Poland, those eligible to join the club have solved a test approved by a Mensa supervisory psychologist with a score placing them in the top 2% of the population. In practice this means they have achieved an IQ equivalent to 130 or more points in Wechsler’s test ([19]; until 2015, the lower limit of 148 points in Catell’s scale [20] was also in effect; the two scores are equivalent). Because of personal data protection, the authors of the study did not get access to the IQ scores of the specific individuals. The data of 96 matching male controls from our database [21] were evaluated.

Both the evaluated Mensa members and the controls were 21–40 years old. Due to the variable size of the CC relative to age, all individuals were additionally divided into two age groups: the younger group of men in the age range of 21–30 years (68 Mensa members, mean age 25.8 years ±2.8 years; 50 controls, mean age 25.6 years; ±3.1 years) and older group of men in the age range of 31–40 years old (45 MENSA members, mean age 34.4 years ±2.7 years; 46 controls, mean age 34.9 years; ±2.4 years).

All the participants reported no history of head injuries, neurological or mental disorders, as well as no alcohol abuse and no use of intoxicants. No changes in brain structures were identified in the MR images acquired. The MRI examination was performed using constant parameters: TR = 3200 ms, TE = 409 ms, 192 slices, slice thickness = 0.9 mm. No contrast agent was administered.

In order to determine the morphology of the CC measurements were performed on T2-weighted images in midsagittal plane.

**Fig. 1:**
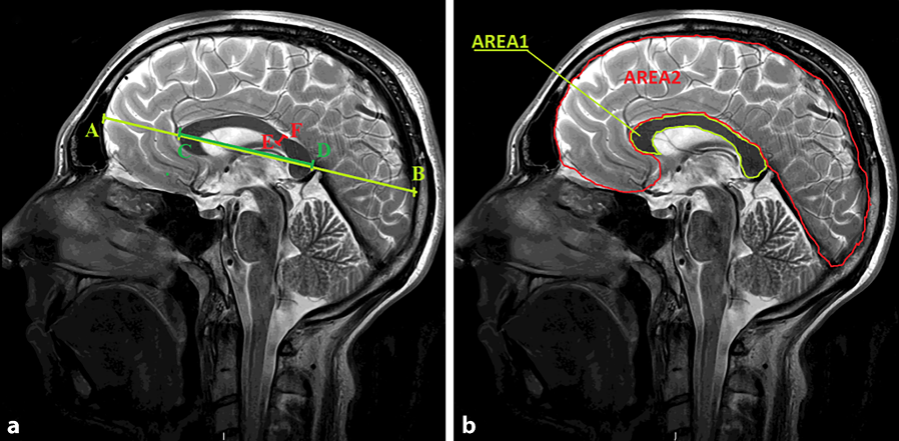
Measurement of **a** corpus callosum dimensions and **b** corpus callosum and brain areas. (symbols explained in the text). *AB* The largest linear dimension of the brain from the frontal lobe to the occipital lobe, *CD* callosal  sectional length, *EF* cross-sectional thickness of the CC in the narrowest location—isthmus, *AREA1* midsagittal callosal cross-sectional area, *AREA2* midsagittal brain cross-sectional area

The following linear measurements were performed (Fig. 1a):

- The largest linear dimension of the brain from the frontal lobe to the occipital lobe (AB)
- Sectional length of the CC (CD)
- Cross-sectional thickness of the CC in the narrowest location—isthmus (EF)

Subsequently, the following areas were measured (Fig. 1b):

- Midsagittal CC cross-sectional area (AREA1)
- Midsagittal brain cross-sectional area, in the plane corresponding to midsagittal CC cross-section (AREA2)

Finally, the ratios of the following specific segments and surface areas were calculated:

- Ratio of the sectional length of the CC and the largest linear dimension of the brain from the frontal lobe to the occipital lobe (CD/AB)
- The ratio of the CC cross-sectional area to the midsagittal brain cross-sectional area (AREA1/AREA2)

The measurements were performed independently by two persons using software dedicated to the morphological measurements—the mean results from the two measurements were calculated. The statistical significance of the differences in the measurements of the CC and the brain between the groups of Mensa members and the respective control groups regarding their age (younger and older groups) was examined using the Analysis of Variance (ANOVA) with Bonferroni correction. The differences between the results were considered to be statistically significant at *p* < 0.05.

## Results

The comparative analyses took into account the specific linear measures as well as the cross-sectional areas of the CC and the brain in the male Mensa members and the participants in the respective control groups, regarding two age groups. The results are shown in Table 1.

**Table 1:** Comparison of linear measures and cross-sectional areas of the corpus callosum and the brain of Mensa members and controls

Groups	Mensa		Controls		Age effect	Group effect	Interaction effect
Age groups	Younger	Older	Younger	Older			
AB (mm)	165.50 ± 0.89	162.89 ± 1.11	159.29 ± 1.07	158.10 ± 1.06	0.07	0.00**	0.49
CD (mm)	72.37 ± 0.49	71.09 ± 0.60	70.00 ± 0.58	70.06 ± 0.57	0.28	0.00**	0.23
EF (mm)	4.44 ± 0.10	4.83 ± 0.12	3.49 ± 0.12	3.62 ± 0.12	0.03*	0.00**	0.27
CD/AB	0.44 ± 0.0029	0.44 ± 0.0035	0.44 ± 0.0034	0.44 ± 0.0034	0.52	0.71	0.12
AREA1 (mm^2^)	648.59 ± 10.49	659.53 ± 12.66	620.22 ± 12.11	629.48 ± 12.11	0.40	0.01*	0.94
AREA2 (mm^2^)	10,113.70 ± 81.75	9782.58 ± 100.45	9782.58 ± 97.30	9833.95 ± 99.39	0.03*	0.46	0.20
AREA1/AREA2	0.06 ± 0.001	0.07 ± 0.001	0.06 ± 0.001	0.06 ± 0.001	0.01*	0.04*	0.20

*AREA1* midsagittal callosal cross-sectional area, *AREA2* midsagittal brain cross-sectional area, *AB* the largest linear dimension of the brain from the frontal lobe to the occipital lobe, *CD* callosal sectional length, *EF* cross-sectional thickness of the CC in the narrowest location—isthmus

*NS* not significant

*Statistically significant at *p* < 0.05; ** statistically significant at *p* < 0.01

Mensa/controls group effect

The comparative analysis showed that the Mensa–control group effect was present for a number of brain and CC parameters with the exception of the whole brain area. The mean length of the CC (CD) and the thickness of the isthmus (EF) were significantly greater in the Mensa male members compared to the control groups. The largest linear dimension of the brain from the frontal lobe to the occipital lobe (AB) was significantly larger in the Mensa members. The mean CC cross-sectional area (AREA1) was significantly greater in the Mensa members. Conversely, no statistically significant differences were identified in the mean brain cross-sectional areas (AREA2). The ratio of the CC cross-sectional area to the brain area (AREA1/AREA2) was significantly higher in the group of Mensa members.

### Age effect

Both in the Mensa groups and the control groups, there was a strong age effect on the cross-sectional thickness of the CC in the narrowest location—isthmus (EF). This distance was significantly bigger in older than in younger individuals. A significant age difference was also visible for the ratio of the CC cross-sectional area to the midsagittal brain cross-sectional area (AREA1/AREA2) and midsagittal CC cross-sectional area (AREA1).

### Interaction of age and Mensa/control group effects

No significant age × MENSA/control group interactions were found for any of the parameters studied (*p* > 0.05).

## Discussion

Many researchers have tried to identify brain structures responsible for intelligence [10, 22–30]. It has also been suggested that human intelligence may be associated with the morphology of the CC [11–15]. The CC is the largest white matter structure in the human brain, consisting of a thick bundle of white fibers [31–33]. The CC forms the major connection between the cerebral hemispheres and is thought to play an important role in interhemispheric communication and control. A number of studies carried out so far have investigated the associations between cognitive capacities and the characteristics of the CC morphology [10, 23, 28, 30]. However, there is insufficient evidence related to individuals presenting above-average intelligence.

The current study was designed to assess the differences in the parameters reflecting the dimensions of the CC between the individuals from the control groups and members of Mensa, characterized with high IQ. The study focused only on male participants from specific age groups because of the results of previous studies on the sex and age effect on CC size. Some studies suggest that there are sexual dimorphisms in the morphology of the CC; however, the related results differ across various research groups [15, 21, 34–37]. There are some studies reporting smaller CC in men than in women [11, 31], but on the other hand some studies indicate the opposite sex effect [34, 37]. An age effect on CC size has also been confirmed [13, 21, 31, 38–41]. Previous research has found that the CC changes in terms of structure throughout life, predominantly during childhood and adolescence [40–42]. In this study, it was found that it becomes thicker in the isthmus between the ages of 21–30 and 31–40.

### Midsagittal cross-sectional dimensions of the corpus callosum

The current study found a significantly wider isthmus of the CC (segment EF) and a longer CC (segment CD) in male members of Mensa compared to the control group.

As reported by Men et al. [8], the overall thickness of Einstein’s CC was greater than the mean thickness of this brain structure in the control groups, one of these comprising 15 older adults (70–80 years of age) and the other consisting of 52 young individuals (24–30 years). The researchers found that, compared to the younger group, Einstein’s CC was significantly longer, while no statistically significant differences were identified compared to the older control group.

An analysis of midsagittal measures of regional callosal thickness in 495 participants, performed by Westerhausen et al. [30], showed a positive association between the splenium of the CC and verbal intelligence as well as performance IQ raw test scores obtained with the Wechsler Adult Intelligence Scale-Revised (WAIS-R). On the other hand, Luders et al. [28] noticed negative correlations between callosal thickness and intelligence tested with the Wechsler Abbreviated Scale of Intelligence (WASI), but this study concerned children and adolescents aged 6–17 years. Likewise, Peterson et al. [11] in a study involving participants aged 6–88 years demonstrated that a thinner and more concave anterior callosal body corresponds to higher IQ.

It is suggested that positive correlations between intelligence and the thickness of the posterior callosal section may reflect a more effective transfer of information between the hemispheres, which enables better processing and integration of the information and consequently higher intellectual capacity [10, 35]. Based on previous studies, it was found that the thickening of the CC is an important feature of development, while its thinning may result from a number of disorders affecting the development or impairing this brain structure [40, 43–45].

### Midsagittal cross-sectional area of the corpus callosum

The midsagittal callosal area is an indicator of the total number of small-diameter fibers in the CC [35]. The present findings showed that the surface area of the CC is greater in the Mensa members compared to the respective control groups. No such relationships were identified in the case of the midsagittal brain cross-sectional area. It may prove the role of CC in intellectual capacity.

Men et al. [8] demonstrated that Einstein’s CC cross-sectional area was significantly greater than in the controls, irrespective of their age. In accordance with the results reported by Millichap et al. [46], a decreased CC cross-sectional area correlated with lower verbal IQ and lower scores in verbal fluency in 72 preterm boys, examined at adolescence (14–15 years of age). Strauss et al. [47] examined 47 patients with epilepsy and 55 normal controls (aged 12–57 years). They found that intellectual capacity, evaluated with the WAIS‑R, was positively related to greater cross-sectional surface area of the posterior callosum. Based on a study by Fine et al. [14], it was suggested that individuals with better reading fluency, achieving higher scores in the WASI test, have a greater cross-sectional surface area in the central segment of the CC compared to the participants with poorer respective results. On the other hand, Hutchinson et al. [13] in a study involving 38 male and 40 female participants (aged 14–25 years) showed that higher IQ on the WASI test was associated with smaller surface area of the posterior CC segments. Likewise, a negative association between the surface area of the CC and intelligence, tested using the Wechsler Intelligence Scale for Children—Revised (WISC-R), was found by Narberhaus et al. [48] in a study involving 64 adolescents born preterm.

### Study limitations

The current study has certain limitations. Due to the participation of a specific group of volunteers, our study concerns only young male members of Mensa International. To strengthen any future analysis, it would be desirable to compare not only these two age groups but to evaluate CC sizes in other age groups of both sexes too. Moreover, it would be interesting to become involved in further studies of adults who had been diagnosed with fetal alcohol syndrome disease (FASD), as FASD is associated not only with cognitive disability, but also with CC volume reduction [49–51]. The further limitation of this study was that due to personal data protection the authors did not acquire the values of the specific participants and thus it was impossible to check the correlation between CC dimensions and particular IQ results. It would also be desirable to expand our Mensa study in the future with other MRI modalities such as functional magnetic resonance imaging (fMRI) and diffusion tensor imaging (DTI) in order to identify and evaluate cognitive possibilities [52, 53] and connectivity within the CC [54–56] as well as magnetic resonance spectroscopy (MRS) to evaluate its metabolism [57].

To the best of the authors’ knowledge, this is the first study comparing the size of the CC between Mensa members and matching control groups. The findings show that the dimensions (linear measures and midsagittal cross-sectional surface area) of this structure were significantly greater in members of Mensa analyzed in this study and may be a basis for further studies about the relationship between intelligence and callosal size in other groups of people as well as using other imaging methods.

## Declarations

All procedures performed in studies involving human participants or on human tissue were in accordance with the ethical standards of the institutional and/or national research committee (Bioethics Commission at Jagiellonian University (approval no. 122.6120.28.2017)) and with the 1975 Helsinki declaration and its later amendments or comparable ethical standards. Informed consent was obtained from all individual participants included in the study.

The supplement containing this article is not sponsored by industry.
